# Characteristics of systemic growth activation by 9,10-ketol-12(*Z*),15(*Z*)-octadecadienoic acid (KODA) in *Populus alba* cultured in vitro

**DOI:** 10.5511/plantbiotechnology.24.0721b

**Published:** 2024-12-25

**Authors:** Mineyuki Yokoyama, Rumi Kaida, Kensuke Miyamoto, Yuichi Tada, Yoshiharu Fujii

**Affiliations:** 1 Department of International Environmental and Agricultural Sciences, Faculty of Agriculture, Tokyo University of Agriculture and Technology, 3-5-8, Saiwai-cho, Fuchu, Tokyo 183-8509, Japan; 2 School of Bioscience and Biotechnology, Tokyo University of Technology, 1404-1, Hachioji, Tokyo 192-0982, Japan; 3 Faculty of Liberal Arts, Science & Global Education, Osaka Metropolitan University, 1-1, Gakuen-cho, Naka-ku, Sakai, Osaka 599-8531, Japan

**Keywords:** internode elongation, KODA, oxylipin, *Populus alba*, premature tissue

## Abstract

α-Ketol octadecadienoic acid (KODA), an oxylipin, is generated from linolenic acid by 9-specific lipoxygenase, while jasmonic acid is ultimately synthesized from the same linolenic acid by 13-specific lipoxygenase. KODA has a unique action different from jasmonic acid, such as promotion of flower formation, activation of rooting, increase of shoot germinating in spring, and breaking endodormancy. We report here that KODA promotes the systemic growth in juvenile *Populus alba* cultured in vitro probably through the activation of immature tissue. Two newly growing shoots emerging from axillary buds of *Populus alba* shoots cultured in vitro, were cut off. One was immersed in 10 µM KODA for 3 min while the other in water as a control. The growth of the plants developing from the shoots was observed one month later. KODA strongly promoted the growth of the primary roots and the aerial parts, in which leaves were mainly contributed. Measurement of the length of each internode revealed that KODA significantly acted on the elongation zone in the stem; clearly extending the length of the second and the third position of internode. The total node number was not significantly different from that in the control. Accordingly, KODA had little effect on the height of the whole shoot. Combined with the previous research of KODA, these findings suggest that KODA application systemically promotes the growth of *Populus alba* cultured in vitro by improving the growth of immature tissues of all organs including roots, stem, and leaves.

α-Ketol octadecadienoic acid (KODA) is a kind of oxylipin having multimodal effects, on flowering in various species, such as *Dianthus caryophyllus* (Yokoyama 2005), *Malus domestica* Borkh (Kittikorn et al. 2010), *Citrus unshiu* Marc (Nakajima et al. 2011), and *Pharbitis nil* (Ono et al. 2013), on rooting in *Zelkova serrata* (Yamamoto et al. 2010) and *Swertia japonica* (Kawakami et al. 2015), and on breaking endodormancy in *Pyrus pyrifolia* Nakai (Sakamoto et al. 2010). The effects on flowering were observed only when the plants were under conditions insufficient for inducing flower bud formation, and conversely, not under the sufficient conditions (Kittikorn et al. 2011; Nakajima et al. 2011; Ono et al. 2013). The stimulatory effects on flowering by KODA were caused by the inhibition of the expression of the suppressing gene for flower-induction, *TERMINAL FLOWER 1* (*TFL1*), not promotion of the flower-inducing gene, *FLOWERING LOCUS T* (*FT*) (Kittikorn et al. 2010; Ono et al. 2013). Nakajima et al. (2011) analyzed in detail how KODA promoted flower induction when applied at the floral inductive stage (in autumn) in *Citrus unshiu* Marc. They counted the number of flower buds and sprouting nodes, and concluded that the principal cause of the increase in flower production by KODA treatment was the promotion of axillary bud sprouting, not limited to flower buds. Furthermore, they also showed that KODA increased the number of spring shoots developing in the following year when applied in summer to the same species (Nakajima et al. 2016). Sakamoto et al. (2012) applied KODA to Japanese pear, *Pyrus pyrifolia* Nakai in early-July and found that KODA increased not only lateral floral buds but also vegetative buds in the apical flower buds, leading to a decrease in the number of blind buds in the next year, which is valuable for the fruit producing industry. The purpose of this research is to investigate the effects of KODA on juvenile plant growth.

We describe here the promoting effects of KODA on the early vegetative growth of *Populus alba* cultured in vitro and discuss the mode of action of KODA on the growth of the plant. Sterile seedlings of *Populus alba* were sub-cultured in half-concentrated Murashige and Skoog (MS) medium (pH 5.7) containing 2% (w/v) sucrose and 0.8% (w/v) agar at 25°C under a cycle of 16-h light (50 µmol m^−2^ s^−1^) and 8-h darkness. Subculture was carried out about once every month by cutting the apical portion off in about 4 cm length, leaving two leaves, from the stalks (or shoots) about 12 cm tall. KODA was prepared using recombinant *E. coli* transgenic for 9-lipoxygenase gene (*LpLOX*) and allene oxide synthase gene (*LpAOS*) of *Lemna paucicostata* SH strain, which is a high producer of KODA (Takagi et al. 2024).

Since the growth rate of the plants developing from the shoots varied greatly with the explant even under the same conditions due to the differences among the individuals, we pinched the shoot tip to induce a pair of new shoots from the same stem. The pair of shoots generated from the same explant was likely to develop at a similar growth rate. One side of the pair (ca. 4 cm) was immediately immersed in a 10 µM KODA solution containing 0.06% ethanol for 3 min and the other side was immersed in 0.06% ethanol as a control.

After KODA treatment, each excised shoot was put in agar and incubated under the same condition that was used for subculture. We repeated the experiments at least ten times and analyzed the data of 4 replicates in the experiments using a pair of new shoots from the same stem. The number and length of primary roots, the number and area of leaves, and the height of shoots were measured using the photographed image of the picture with ImageJ software (National Institutes of Health (open source), freeware image analysis, v1.52). After weighing the aerial parts, we carefully cut off the secondary roots emerging from the primary roots and measured the weight of the primary roots and the secondary roots separately. The length of each internode in the aerial parts was also measured to determine the region affected by KODA treatment. All leaves in the aerial parts were removed. The remaining stems were immersed in heated ethanol at 60°C for 10 min and stocked in a refrigerator until measurement of internode length. The length of each internode was measured on the enlarged image of the photographs.

KODA prominently activated the entire growth of *Populus alba* cultured in vitro one month after treatment (Figure 1). KODA treatment stimulated the growth of the primary roots, that is, fresh weight, root number, root length and thickness one month after treatment, but not that of the secondary roots, derived from the primary roots (Figure 2). The lack of effect on the secondary roots may be attributed to the observation period. We analyzed the root growth one month after KODA treatment. The effect on the secondary roots may have been detectable at a later date. The fresh weight of the aerial parts was also greatly increased with leaf area being much larger in the KODA-treated group than in the control. On the other hand, the leaf number was not significantly different (Figure 3). It is intriguing that KODA elongated only the second and third internode, that is, the latter part of the extension zone (Figure 4A). The extension zone exists below the shoot tip to above the mature tissue having a hard cell wall. The extension zone is divided into the accelerating part, where the lower cells have a higher growth rate, and decelerating part, where the lower cells have a lower growth rate (Pritchard 1994). KODA seems to expand the decelerating zone in the extension zone. There was no difference in the number of internodes between the control and KODA experiment (Figure 4). KODA had only a marginal effect on the total height (shoot length) at *p*=0.058 (Figure 4). This is in agreement with the fact that the effect of KODA on the shoot was only promotion of elongation in the extension zone. The slightly smaller number of leaves (Figure 3) as compared to the number of internodes (Figure 4C) was caused by the difference in the measurement method. The internode number was determined from the enlarged image of the photographs while the leaf number was counted with the naked eye. Leaves surrounding the apex were too small to separate with the naked eye.

**Figure 1:**
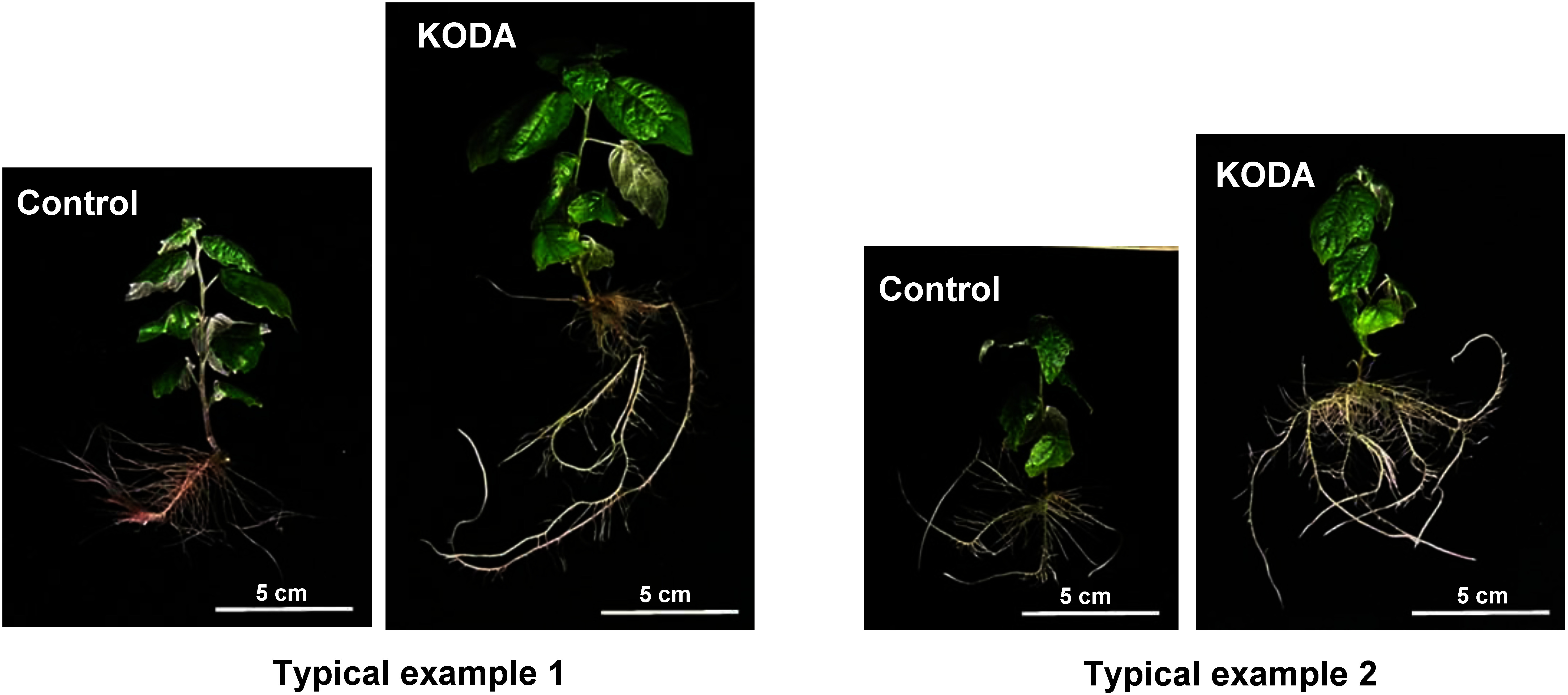
Figure 1. Typical figures of in vitro cultured *Populus alba*. The pair of shoots were cut off at the same time from the same individual. Four sets of pairs, after immersed in 10 µM KODA for just 3 min or water as a control, were incubated for one month. We repeated the experiments more than ten times and analyzed the data of 4 replicates. The pictures show two typical results.

**Figure 2:**
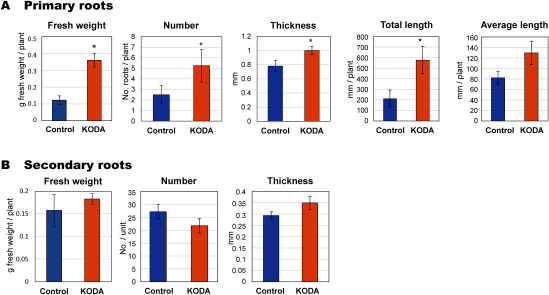
Figure 2. The growth of the roots of *Populus alba* incubated for one month. The growth of the primary roots, that is, the adventitious roots emerged from the excised shoots, is presented as fresh weight, root number, root thickness, total root length and average root length. The growth of the secondary roots that emerged from the primary roots is presented as fresh weight, root number and root thickness. Others are the same as in Figure 1. Asterisk* indicates significant difference at *p*<0.05. *n*=4.

**Figure 3:**
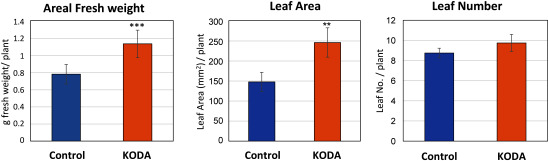
Figure 3. The growth of the aerial parts of *Populus alba* incubated for one month. The number and area of leaves were measured using the photographed image with ImageJ software. Leaf area was the sum of all leaves in each individual and the average value of four individuals is shown. Others are the same as in Figure 1. Asterisk*** indicates significant difference at *p*<0.005 with the asterisk** at *p*<0.01. *n*=4.

**Figure 4:**
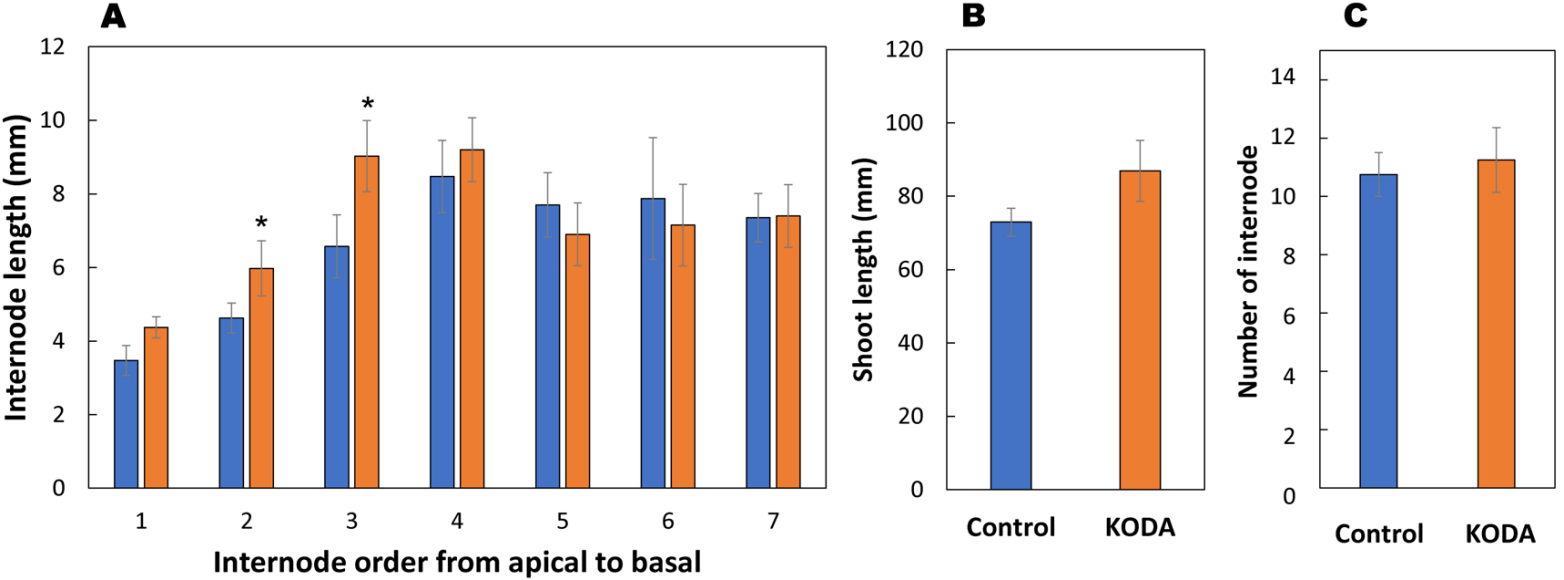
Figure 4. The length of each internode of *Populus alba* incubated for one month. The stems, after all leaves were removed, were immersed in heated ethanol at 60°C for 10 min and stocked in a refrigerator until use. The internode was measured on the enlarged image of the pictures. Others are the same as in Figure 1. Asterisk* indicates significant difference at *p*<0.05. *n*=4.

As described above, KODA notably promoted the early growth of *Populus alba* in in vitro culture systemically over roots, stem and leaves. KODA treatment increased the number, total length and thickness of the primary roots (Figures 1 and 2). KODA also promoted the internode elongation of the stem only in the extension zone (Figure 4). This promotion of the length in internode does not look like auxin or gibberellin effects. The stimulation of elongation by auxin has been intensively studied using stem explants (Cleland 1995). Auxin activates the plasma membrane H^+^-ATPase by phosphorylation (Takahashi et al. 2012), which ultimately results in the promotional elongation of stem segments referred to as ‘acid-growth’ (Kutschera 1994). Expansins, involved in this process, loosen the cell wall, which is a prerequisite for cell expansion (Cosgrove 2000). However, promotion of stem elongation by auxin has been found in experiments using stem explants, but not intact stems like the ones we used in the present study. The effects of auxin on stem segments are related to how auxin loosens the hard cell wall of the mature stem. On the other hand, gibberellin is well known to regulate the growth of the intact stem. Gibberellin extends the stem mainly in intact plants with stretching internodes rather than increasing the number of nodes. Nagai et al. (2020) elucidated that the cell division on the intercalary meristematic region was promoted with gibberellin, leading to internode elongation in the presence of gibberellin. Unfortunately, the intercalary meristem is seen only in monocotyledonous plants. As described above, it is unlikely that KODA acts like auxin or gibberellin. However, there remains the possibility that KODA modifies the plant hormone metabolism including auxin and gibberellin. It is important to follow the hormone changes along time after KODA treatment.

To our knowledge, no chemicals that exert such effects only on the extension zone have been reported. The characteristics of extension zone have been studied mainly using the primary root tissues of maize (*Zea mays*) because the structure of the tissue is not as complicated as that of the stem. The extension zone is naturally divided into accelerating and decelerating regions (Pritchard 1994) as described in the results of Figure 4A. Water deficit induced progressive inhibition of the extension in the decelerating region through increasing lignin metabolism and accumulating wall phenolics (Fan et al. 2006) but the elongation rate in the accelerating region was not affected (Sharp et al. 1988). The position of internode affected by KODA shows that KODA possibly promoted the extension in the decelerating region (Figure 4A). KODA might suppress the process to maturation of cauline cells because the decelerating phase in extension zone is a previous step for maturation. Kawakami et al. (2015) observed that KODA activated the growth of roots generated from *Swertia japonica* calli, which probably included many primordia of roots. Surprisingly, KODA suppressed the maturation of the roots. The secondary metabolite, xanthone diglycoside, was strongly inhibited to deposit with KODA. As described above, water deficit stress induces progressive inhibition of the extension in the decelerating region through hardening cell wall (Fan et al. 2006). These results may suggest that KODA treatment maintains immaturity in premature tissue.

KODA treatment also promotes the enlargement of the leaves of *Populus alba* in our study (Figures 1 and 3) even though we have never noticed the change of leaf area with KODA treatment in the tests in the green house or field. The leaves of plants cultured in vitro are known to be disordered (Hazarika 2006). In in vitro culture, the media containing a high sucrose and salt, low light level and limited carbon dioxide concentration in the air in the culture vessel and high humidity, such factors influence photosynthesis of the plants in vitro culture (Fujiwara and Kozai 1995; Jeong et al. 1995). Although plants cultured in vitro have green leaves and appear normal, photosynthesis (carbon fixation) hardly occurs (Grout and Donkin 1987). In acclimatization, normal photosynthesis is acquired after new leaves appear (Van Huylenbroeck et al. 2000). Stomatal development is also disordered. Although the number of stomata is not changed, the majority of stomata on the surface of plants cultured in vitro do not close in response to abscisic acid or darkness (Hazarika 2006). The cuticle on the surface of the leaves cultured in vitro is also incomplete compared with the leaves grown in a greenhouse (Hazarika 2006). Since the immature leaves easily wilt after being detached from the parent plant, those characteristics seem to be related with the immaturity of the leaves cultured in vitro. The leaves of plants cultured in vitro are reported to be much smaller than those of the plants grown in a field (Pospisilova et al. 1999). This phenomenon also does not contradict with the view that the plants grown in vitro might be forcibly maintained to retain their immature characteristics.

## Abbreviations

KODA: 9-hydroxy-10-oxo-12(*Z*),15(*Z*)-octadecadienoic acid
